# Proteasomal regulation of ASK family kinases dictates cell fate under hyperosmotic stress

**DOI:** 10.1016/j.jbc.2025.110566

**Published:** 2025-08-06

**Authors:** Xiangyu Zhou, Kengo Watanabe, Kazuhiro Morishita, Jun Hamazaki, Shigeo Murata, Isao Naguro, Hidenori Ichijo

**Affiliations:** 1 Laboratory of Cell Signaling, Graduate School of Pharmaceutical Sciences, The University of Tokyo, Tokyo, Japan; 2 Laboratory of Protein Metabolism, Graduate School of Pharmaceutical Sciences, The University of Tokyo, Tokyo, Japan

**Keywords:** apoptosis signal-regulating kinase 3, apoptosis signal-regulating kinase 1, proteasome, proteotoxicity, hyperosmotic stress, high-content genome-wide siRNA screening

## Abstract

The proteasome has an essential role in proteostasis maintenance and is critical for cell survival under proteotoxic conditions, including hyperosmotic stress. However, how proteasome activity is linked to cell survival/death under hyperosmotic stress is unclear. We previously reported that apoptosis signal–regulating kinase 3 (ASK3) contributes to cell survival through its inactivation under hyperosmotic stress. The 19S regulatory particle subunits of the proteasome were enriched in the ASK3 inactivator candidates identified through our genome-wide siRNA screening. In this study, we demonstrated that the proteasome regulates ASK3 inactivation through its proteolytic activity. Intriguingly, the proteasome inactivates ASK3 *via* degradation of not ASK3 *per se* but another ASK family member, ASK1, which activates ASK3 in a kinase activity–dependent manner. Furthermore, elevated ASK1 levels under proteasome inhibition sensitize cells to hyperosmotic stress. These findings suggest that ASK family kinases function as key mediators linking proteasome capacity to cellular susceptibility under hyperosmotic stress.

Cells are separated from the outside environment by the plasma membrane. Since it is impermeable to diverse biomolecules and has a relatively high permeability to water (1), the osmotic balance between the intracellular and extracellular space is critical for water flux across the plasma membrane. When the extracellular osmolality is higher than the intracellular osmolality, water flows from the intracellular space to the extracellular space, resulting in cell shrinkage. This condition, hereinafter referred to as hyperosmotic stress, has deleterious effects on the structure and function of biomolecules, organelles, and the cytoskeleton (2). Upon hyperosmotic stress, cells rapidly induce a volume recovery system known as regulatory volume increase (RVI), but excessive and persistent cell shrinkage leads to apoptosis (3, 4).

Cell shrinkage under hyperosmotic stress leads to increases in intracellular ionic strength and macromolecular crowding (3), which can create an imbalance in protein homeostasis (*i.e.*, proteostasis). *In vitro* studies have suggested that increased ionic strength changes the stability of protein structures and decreases protein activity (5, 6, 7) and that increased macromolecular crowding accelerates the aggregation of partially unfolded proteins (8, 9, 10). Consistently, hyperosmotic stress induces protein misfolding and aggregation in intact cells (11, 12). These reports suggest that hyperosmotic stress increases the burden on the processes that degrade abnormal intracellular proteins, such as the proteasome and autophagy. In addition, disruption of the proteasome or autophagy decreases the survival of *Caenorhabditis elegans* and mammalian cells under hyperosmotic stress (13, 14, 15), suggesting that the integrity of proteostasis machinery is a key factor for the survival of cells facing proteotoxicity under hyperosmotic stress. However, the molecular mechanisms that link cellular proteostasis machinery overload to cell survival under hyperosmotic stress have not been identified.

Previously, we found that apoptosis signal–regulating kinase 3 (ASK3; also known as mitogen-activated protein kinase kinase kinase 15 [MAP3K15]) is rapidly inactivated upon hyperosmotic stress (16) and that its inactivation is important for RVI and cell survival under hyperosmotic stress (17). By leveraging a genome-wide siRNA screening, we have reported upstream regulators of ASK3 inactivation including protein phosphatase 6 (PP6), a direct phosphatase of ASK3, and nicotinamide phosphoribosyltransferase (NAMPT), which regulates the PP6–ASK3 interaction under hyperosmotic stress (17, 18). Moreover, through the screening, we found that many proteasome-related genes were predicted to be ASK3 inactivator candidates.

In the present study, we report that the proteasome is a regulator of ASK3 inactivation under hyperosmotic stress. We demonstrate that proteasome activity decreases the protein level of another ASK family member, ASK1 (19), thereby suppressing the inhibitory effect of ASK1 on ASK3 inactivation. Furthermore, ASK1 mediates the acceleration of hyperosmotic stress–induced apoptosis *via* proteasome inhibition. Our results suggest that proteasomal regulation of ASK1 protein levels controls ASK3 signaling and cell death under hyperosmotic stress, revealing the role of ASK family proteins as linking molecules between proteostasis capacity and cell fate under hyperosmotic stress.

## Results

### The proteasome negatively regulates ASK3 activity under hyperosmotic stress

**Figure 1:**
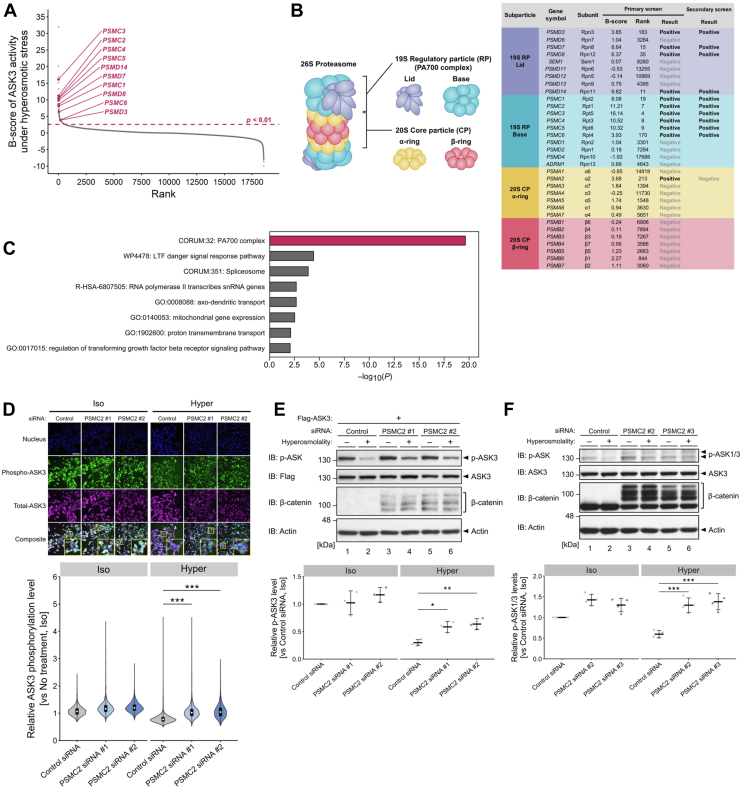
**The proteasome is a regulator of ASK3 inactivation under hyperosmotic stress.***A*, a rank-ordered plot of *B*-scores for all genes subjected to the primary screening procedure (17). Each gene is represented as a *point* on the plot. A gene with a higher *B*-score corresponds to a stronger ASK3 inactivator candidate. The 19S proteasome subunit genes that were positive on the screening are highlighted in *magenta*. *B*, scores, ranks, and results for proteasome subunit genes in the genome-wide siRNA screening. *C*, Gene Ontology (GO) terms enriched in the positive genes identified in the second screening. Terms satisfying the default criteria of Metascape analysis (38) and their nominal *p* values are presented. The 19S RP (PA700 complex) is highlighted in *magenta*. *D*, effect of PSMC2 depletion on ASK3 phosphorylation level in tetracycline-inducible Flag-ASK3-stably expressing cells. The *top panel* shows immunofluorescence images of nuclei, phospho-ASK3, total-ASK3, and their combination with enlarged images of the square region. The *bottom panel* shows violin plots of ASK3 phosphorylation level in each cell, which was defined as the relative fluorescence intensity of phospho-ASK3 to total ASK3. Control siRNA: *n* = 1065 cells (iso), 1493 cells (hyper); PSMC2 siRNA #1: *n* = 1943 cells (iso), 2234 cells (hyper); and PSMC2 siRNA #2: *n* = 2000 cells (iso), 2293 cells (hyper). Representative data from three independent experiments are shown. The *white scale bar* represents 200 μm. *E* and *F*, effects of PSMC2 depletion on the phosphorylation level of exogenously expressed ASK3 (*E*) or endogenous ASK3 (*F*) under hyperosmotic stress. The accumulation of ubiquitinated β-catenin is an indicator of proteasome dysfunction because of PSMC2 depletion. The *bottom graphs* depict the quantification of the Western blot data. Individual values and the mean ± SD are presented as *point*s and *bars*, respectively. *n* = 3 (*E*) and 4 (*F*) biological replicates. *D*–*F*, iso, 300 mOsm; hyper, 400 mOsm; 10 min. Statistical analyses were performed using Kruskal‒Wallis test followed by Dunn’s test (*D*) and Dunnett’s test (*E* and *F*). ∗*p* < 0.05, ∗∗*p* < 0.01, and ∗∗∗*p* < 0.001. See also Figure S1. ASK3, apoptosis signal–regulating kinase 3; IB, immunoblotting; RP, regulatory particle.

In our previous study, a genome-wide siRNA screening was conducted to comprehensively identify the negative regulators of ASK3 activity under hyperosmotic stress. This screening utilized ASK3 phosphorylation level, determined by immunofluorescence-based high-content analysis, as a readout and identified 63 positive genes (17) (Fig. S1, *A* and *B*). Among these positive genes, many proteasome subunit genes were included (Fig. 1, *A* and *B*). We then performed functional enrichment analysis of the positive genes and found that genes encoding the 19S regulatory particle (RP; PA700 in Fig. 1B) of the proteasome were significantly enriched in the positive genes (Figs. 1, *B* and *C*, and S1*C*). To validate the screening results, we knocked down the positive genes encoding the 19S RP subunits and examined the effects on ASK3 activity using a phospho-specific antibody that recognizes Thr808 of ASK3, whose phosphorylation is critical for its kinase activity (16). Depletion of the proteasome 26S subunit ATPase 2 (PSMC2) upregulated the phosphorylation level of exogenously expressed ASK3 (p-ASK3) under hyperosmotic stress, as detected by immunofluorescence (Fig. 1*D*). The upregulation of p-ASK3 level under hyperosmotic stress by PSMC2 depletion was also confirmed by immunoblotting for both exogenously expressed ASK3 (Fig. 1*E*; lanes 2, 4, and 6) and endogenous ASK (Fig. 1*F*; lanes 2, 4, and 6). Note that the phospho-specific antibody for ASK3 also recognizes the phosphorylated form of other ASK family proteins (20) and reflects overall ASK activity derived from ASK1 and ASK3 because of their similar and relatively high molecular weights (ASK1: 155 kDa, ASK3: 147 kDa), particularly according to endogenous-level immunoblotting evaluations (*e.g.*, Fig. 1*F*). Depletion of another 19S RP subunit, PSMC3, also resulted in increased endogenous p-ASK1/3 levels in hyperosmotic stress (Fig. S1*D*). These results suggest that the proteasome suppresses ASK3 activity under hyperosmotic stress.

### Proteasome activity is required for the maintenance of low ASK3 activity under hyperosmotic stress

**Figure 2:**
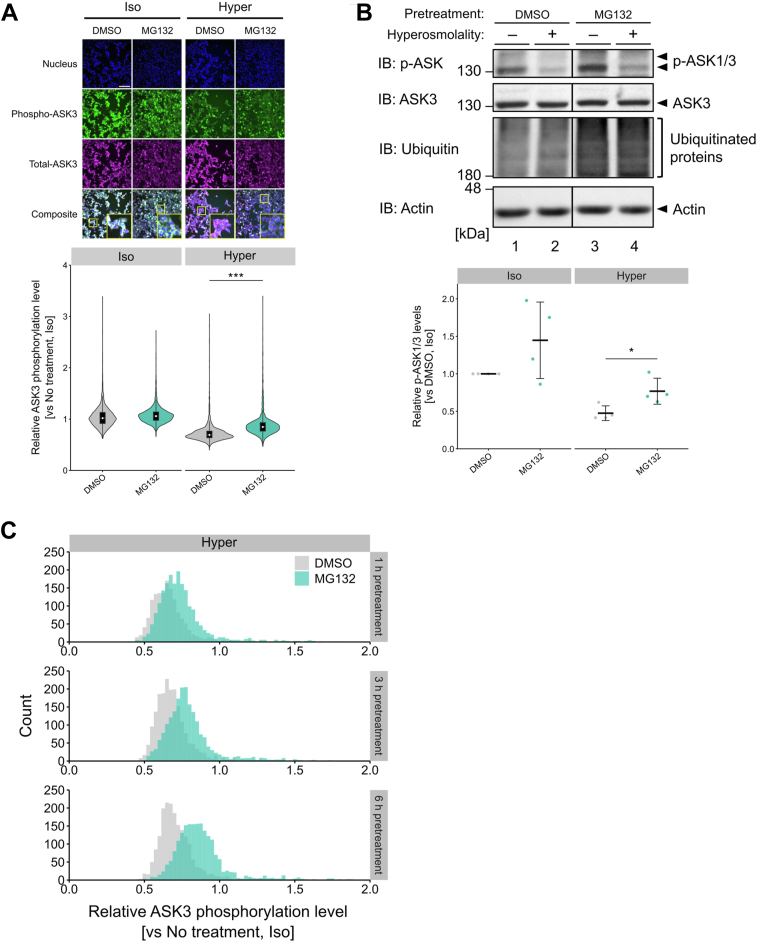
**Proteasome activity is required for the suppression of ASK3 activity under hyperosmotic stress.***A*, effect of MG132 on ASK3 phosphorylation level in tetracycline-inducible Flag-ASK3-stably expressing cells. Cells were pretreated with 10 μM MG132 for 6 h before exposure to osmotic stimuli. ASK3 phosphorylation level was measured as described in Figure 1*D*. The *top panel* shows immunofluorescence images of nuclei, phospho-ASK3, total-ASK3, and their combination with enlarged images of the square region. The *bottom panel* shows violin plots of ASK3 phosphorylation level in each cell. DMSO: *n* = 1876 cells (iso), 1900 cells (hyper); MG132: *n* = 2280 cells (iso), 1991 cells (hyper). Representative data from four independent experiments are shown. The *white scale bar* represents 200 μm. *B*, effects of MG132 on endogenous p-ASK1/3 levels. Cells were pretreated with 10 μM MG132 for 4 h before exposure to osmotic stimuli. The efficacy of proteasome inhibition was confirmed by the accumulation of ubiquitinated proteins. The *bottom panel* shows the quantification of the Western blot data. Individual values and the mean ± SD are presented as *points* and *bars*, respectively. *n* = 4 biological replicates. *C*, time dependency of the effects of MG132 on ASK3 phosphorylation level under hyperosmotic stress. Tetracycline-inducible Flag-ASK3-stably expressing cells were pretreated with 10 μM MG132 for 1, 3, or 6 h before exposure to osmotic stimuli. ASK3 phosphorylation level was measured as described in Figure 1*D*. Histograms of ASK3 phosphorylation level in each cell are shown. Outlier cells with relative ASK3 phosphorylation level greater than two are not shown. DMSO: *n* = 1451 cells (1 h), 1910 cells (3 h), and 1900 cells (6 h); MG132: *n* = 1964 cells (1 h), 1970 cells (3 h), and 1991 cells (6 h). Representative data from two independent experiments are shown. *A*–*C*, iso, 300 mOsm; hyper, 400 mOsm; 10 min. Statistical analyses were performed using Wilcoxon rank sum test (*A*) and unpaired two-tailed Student’s *t* test (*B*). ∗*p* < 0.05, ∗∗∗*p* < 0.001. See also Figure S2. ASK3, apoptosis signal–regulating kinase 3; BTZ, bortezomib; DMSO, dimethyl sulfoxide; IB, immunoblotting.

The proteasome is composed of two subcomplexes: the catalytic 20S core particle (CP) and the 19S RP. In the process of ubiquitin-dependent degradation, the 19S RP recognizes, unfolds, deubiquitinates, and translocates ubiquitinated substrates to the 20S CP where proteolysis takes place (21). Interestingly, all proteasome-related genes included in the positive genes were those encoding 19S RP subunits (Fig. 1*B*). We then investigated whether the catalytic activity of the proteasome is required for ASK3 inactivation under hyperosmotic stress. Treatment with MG132 and bortezomib, proteasome inhibitors that block the catalytic activities of the 20S CP subunits (22), caused significant increases in ASK3 phosphorylation under hyperosmotic stress (Figs. 2*A* and S2*A*). Treatment with proteasome inhibitors increased endogenous p-ASK1/3 levels under iso-osmotic and hyperosmotic condition (Figs. 2*B* and S2*B*). Moreover, siRNA-mediated depletion of the 20S CP subunit PSMA1 also increased p-ASK1/3 levels (Fig. S2*C*), indicating that 20S CP genes were false negatives in our screening, presumably because depletion of the 20S CP subunit was severely toxic to cells, and only cells with low knockdown efficiency survived. These results suggest that the maintenance of low ASK3 activity under hyperosmotic stress requires the proteolytic activity of the proteasome.

The MG132 treatment-induced increase in ASK3 phosphorylation level under hyperosmotic stress (Fig. 2*A*) increased as the duration of MG132 pretreatment increased from 1 h to 6 h (Fig. 2*C*). The proteasome activity decreased to approximately 10% at 1 h after inhibitor treatment and remained almost constant until 6 h (Fig. S2*D*). These results implied that the time-dependent effect of MG132 on ASK3 phosphorylation is not because of a time-dependent decrease in proteasome activity but rather the time-dependent accumulation of a certain proteasome substrate before exposure to hyperosmotic stress. Taken together, these results suggest that degradation of proteasome substrates at steady state is required for maintaining ASK3 in an inactivated state under hyperosmotic stress.

### Increased ASK1 protein levels disturb ASK3 inactivation under proteasome inhibition

**Figure 3:**
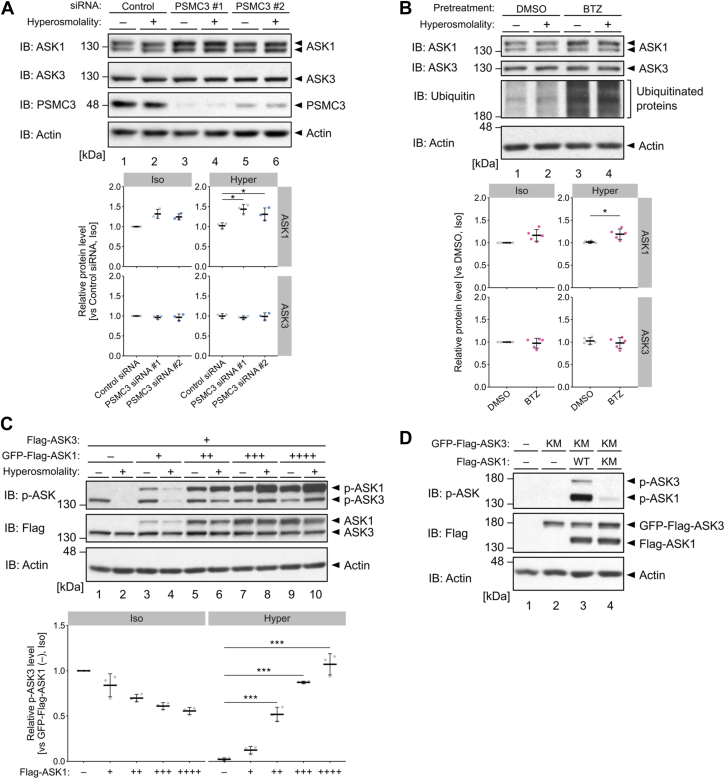
**Increased ASK1 protein levels disturb ASK3 inactivation under proteasome inhibition.***A*, effect of PSMC3 depletion on the protein levels of ASK1 and ASK3. *B*, effects of proteasome inhibition on the protein levels of ASK1 and ASK3. Cells were pretreated with 100 nM BTZ for 6 h before osmotic stimuli. Proteasome inhibition was confirmed by the accumulation of ubiquitinated proteins. *C*, effects of ASK1 protein levels on ASK3 phosphorylation level under hyperosmotic stress. Flag-ASK3 and GFP-Flag-ASK1 were exogenously expressed in cells. *D,* transphosphorylation of ASK3 by ASK1. GFP-Flag-ASK3 and Flag-ASK1 were exogenously expressed in cells. *A*–*C*, the *bottom panels* show the quantification of the Western blot data. Individual values and the mean ± SD are presented as *points* and *bars*, respectively. *n* = 3 (*A*, *C*, *D*) and 5 (*B*) biological replicates. Iso, 300 mOsm (*A*–*C*); hyper, 400 mOsm (*A* and *B*) or 500 mOsm (*C*); 10 min. Statistical analyses were performed using Dunnett’s test (*A* and *C*) and unpaired two-tailed Student’s *t* test (*B*). ∗*p* < 0.05, ∗∗∗*p* < 0.001. See also Figure S3. ASK 1/3, apoptosis signal–regulating kinase 1/3; BTZ, bortezomib; IB, immunoblotting; KM, kinase-negative mutant; WT, wildtype.

We next searched for the proteasome substrate whose degradation is required for ASK3 inactivation. Since the inhibition of protein synthesis by cycloheximide did not decrease the intracellular protein level of ASK3 under iso-osmotic and hyperosmotic condition within 1 h (Fig. S3*A*), it is unlikely that hyperosmotic stress-induced ASK3 inactivation stemmed from the proteasomal degradation of phosphorylated ASK3 (*i.e.*, active form) followed by its replacement with newly synthesized ASK3 (*i.e.*, inactivated form). In contrast to that of ASK3, the protein level of ASK1 was increased when the proteasome subunit PSMC3 was depleted (Fig. 3*A*) or when proteasome activity was inhibited by bortezomib treatment (Fig. 3*B*). ASK1 mRNA levels did not increase during bortezomib treatment (Fig. S3*B*), implying that the increase in the ASK1 protein level induced by proteasome inhibition was not because of the transcriptional regulation of ASK1.

It has been reported that ASK1 forms a heteromeric complex with another ASK family member, which determines basal ASK1 kinase activity *via* autophosphorylation and transphosphorylation (23). Given that ASK1 also forms a complex with ASK3 (24), we hypothesized that proteasome inhibition at steady state increases the ASK1 protein level and that the transphosphorylation of ASK3 by the upregulated ASK1 is responsible for the increases in ASK3 activity under hyperosmotic stress. We therefore investigated the effect of the ASK1 protein level on the activity of ASK3 under hyperosmotic stress. To control and evaluate the activities of ASK1 and ASK3 individually, we utilized GFP as a conjugating tag in an ASK1–ASK3 coexpression immunoblotting system in which ASK3 was clearly distinguished from ASK1 by molecular weight. An increase in the ASK1 protein level abrogated ASK3 inactivation, and the highest ASK1–ASK3 ratio even caused ASK3 activation under hyperosmotic stress (Fig. 3*C*). This effect of ASK1 on ASK3 phosphorylation level was dependent on the kinase activity of ASK1 because the phosphorylation of Thr808 in the ASK3 kinase–negative mutant, which lacks autophosphorylation ability (16), was increased by the coexpression of wildtype ASK1 but not by that of ASK1 kinase-negative mutant (25) (Fig. 3*D*). Therefore, these results suggest that the proteasome inhibits the ASK1-mediated phosphorylation of ASK3 *via* degradation of ASK1 to ensure ASK3 inactivation under hyperosmotic stress.

### Proteasome inhibition disturbs ASK3 signaling under hyperosmotic stress in an ASK1-dependent manner

**Figure 4:**
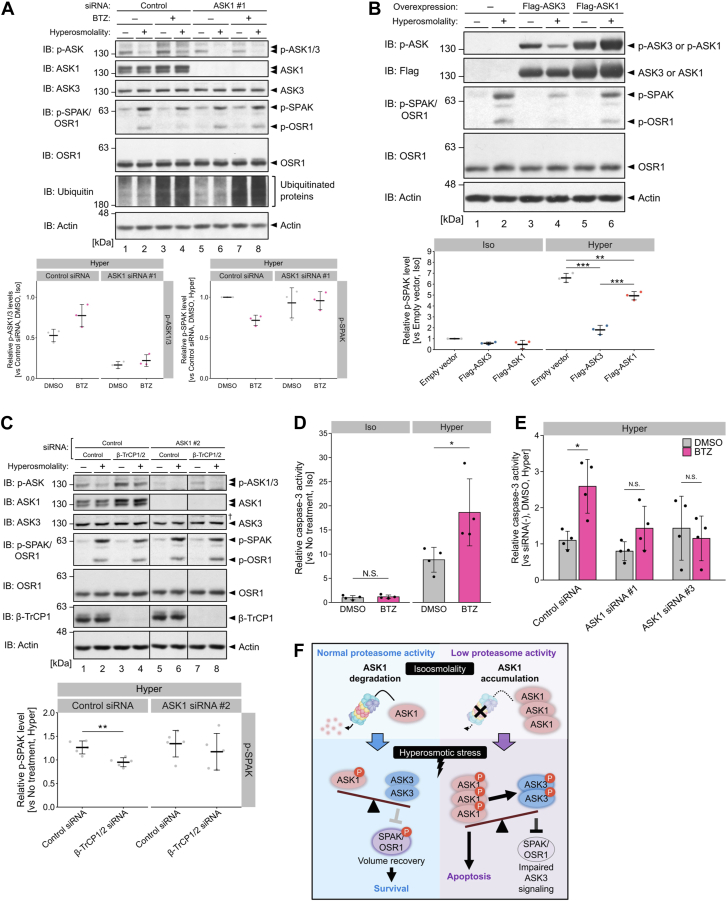
**Proteasome inhibition suppresses the activation of the ASK3 downstream signaling and exacerbates apoptosis under hyperosmotic stress in an ASK1-dependent manner.***A*, requirement of ASK1 for the effects of proteasome inhibition on SPAK/OSR1 phosphorylation levels under hyperosmotic stress. Cells were pretreated with 100 nM BTZ for 6 h before osmotic stimuli. *B*, eeffects of ASK1/3 overexpression on SPAK/OSR1 phosphorylation levels under hyperosmotic stress. *C*, requirement of ASK1 for the effects of β-TrCP1/2 depletion on SPAK/OSR1 phosphorylation levels under hyperosmotic stress. *D*, effect of proteasome inhibition on caspase-3 activation under hyperosmotic stress. *E*, effect of ASK1 depletion on BTZ-induced overactivation of caspase-3 under hyperosmotic stress. *F*, schematic representation of the main findings in this study. *D* and *E*, cells were pretreated with 100 nM BTZ for 6 h, after which the culture medium was changed to BTZ-free osmotic medium. Mean ± SD. *n* = 4 (*D* and *E*) biological replicates. *A*–*C*, the *bottom panels* show the quantification of the Western blot data. Individual values and the mean ± SD are presented as *points* and *bars*, respectively. *n* = 3 (*A* and *B*) and 5 (*C*) biological replicates. Iso, 300 mOsm (*A*–*C*) or culture medium (*D*); hyper, 400 mOsm (*A*–*C*) or culture medium supplemented with 500 mM mannitol (*D* and *E*); 10 min (*A*–*C*) or 12 h (*D* and *E*). Statistical analyses were performed using Tukey–Kramer’s test (*B*, *D*, and *E*) and unpaired two-tailed Student’s *t* test (*C*). ∗*p* < 0.05, ∗∗*p* < 0.01, and ∗∗∗*p* < 0.001. ASK 1/3, apoptosis signal–regulating kinase 1/3; BTZ, bortezomib; NS, not significant; SPAK/OSR1, STE20/SPS1-related proline/alanine-rich kinase/oxidative stress–responsive kinase 1; β-TrCP1/2, beta-transducin repeat containing protein 1/2.

Under hyperosmotic stress, ASK3 inactivation enables the activation of downstream kinase cascades, including with-no-lysine (K) kinase 1 (WNK1), STE20/SPS1-related proline/alanine-rich kinase (SPAK), and oxidative stress–responsive kinase 1 (OSR1) cascades (16). We then examined whether the proteasomal regulation of ASK1 is involved in ASK3-WNK1-SPAK/OSR1 signaling under hyperosmotic stress. ASK1 depletion tended to diminish the bortezomib-induced increase in p-ASK1/3 level under hyperosmotic stress (Fig. 4*A*; lanes 6 and 8), although ASK1 depletion also reduced basal ASK3 phosphorylation level (lanes 5 and 7 in Fig. 4*A*), potentially making the detection range too small to evaluate the effects of bortezomib on ASK3 activity. In addition, pretreatment with bortezomib inhibited hyperosmotic stress–induced SPAK/OSR1 activation in control cells but not in ASK1-knockdown cells (Fig. 4*A*; lanes 2, 4, 6, and 8). These results suggest that ASK1 is responsible for the increased phosphorylation of ASK3 under proteosomal inhibition. In the absence of ASK1, p-ASK3 level under hyperosmotic stress is not affected by bortezomib. Moreover, ASK3 overexpression had a much stronger inhibitory effect on SPAK/OSR1 activation under hyperosmotic stress than ASK1 overexpression (Fig. 4*B*), further supporting that it was not ASK1 that directly inhibited hyperosmotic stress–induced SPAK/OSR1 activation during proteasome inhibition but rather ASK1-transactivated ASK3. Given the potentially enormous effects of proteasome inhibition on a broad spectrum of intracellular signaling pathways, we alternatively induced the accumulation of ASK1 by depleting the beta-transducin repeat containing proteins 1 and 2 (β-TrCP1 and 2), which are E3 ubiquitin ligases for ASK1 (26). β-TrCP1/2 depletion increased ASK1 protein levels but not ASK3 protein levels and inhibited ASK inactivation and SPAK activation under hyperosmotic stress (Fig. 4*C*; lanes 2 and 4). The effects of β-TrCP1/2 depletion on SPAK activation were abrogated by additional depletion of ASK1 (Fig. 4*C*; lanes 4 and 8), suggesting that the increase in the ASK1 protein level induced by β-TrCP1/2 depletion impaired SPAK activation under hyperosmotic stress. Taken together, these results suggest that the proteasome regulates ASK1 protein levels to support ASK3 signaling under hyperosmotic stress.

### Proteasome inhibition causes ASK1-mediated exacerbation of apoptosis under hyperosmotic stress

Since ASK1 is well known to induce apoptosis under various stresses (27), we hypothesized that proteasomal maintenance of ASK1 protein levels is a mechanism linking proteostasis overload and cell fate under hyperosmotic stress. We first tested whether disruption of proteasome function decreases the survival of mammalian cells under hyperosmotic stress. We pretreated cells with bortezomib and changed the culture medium to either iso-osmotic or hyperosmotic buffer without bortezomib. This transient bortezomib pretreatment did not activate caspase-3 in iso-osmotic buffer but accelerated caspase-3 activation under hyperosmotic stress (Fig. 4*D*), demonstrating that the precondition of proteasome inhibition made cells prone to hyperosmotic stress–induced apoptosis. We then tested whether ASK1 is required for the exacerbation of hyperosmotic stress-induced apoptosis by proteotoxic preconditioning. ASK1 knockdown reduced the effect of proteasome inhibition on caspase-3 activity under hyperosmotic stress (Fig. 4*E*). Therefore, proteasome inhibition accelerates apoptosis under hyperosmotic stress in an ASK1-dependent manner.

## Discussion

Although the importance of proteostasis maintenance in the survival of *C. elegans* and mammalian cells under hyperosmotic stress has been reported (13, 14, 15), it has been unknown whether particular molecules sense the integrity of cellular proteostasis pathways to regulate cell survival and death responses under hyperosmotic stress. Here, we demonstrated that insufficient proteasome activity resulted in the accumulation of ASK1 at steady state, which led to the failure of ASK3 signaling and the exacerbation of apoptosis under hyperosmotic stress (Figs. 3 and 4). Given that the proteasome is one of the major degradation machineries for damaged proteins, ASK1-dependent regulation of apoptosis can contribute to the elimination of the cells that are prone to retaining damaged proteins because of low proteasome activity under hyperosmotic stress. Proteasome inhibition also sensitizes cells to other proteotoxic stresses, such as oxidative stress and heat shock, *via* unknown mechanisms (28, 29, 30). Degradation of ASK1 by the ubiquitin‒proteasome system has been reported to prevent cell death under oxidative stress (26, 31, 32, 33). Therefore, it is possible that the proteasomal maintenance of ASK1 protein levels controls the fate of cells under diverse proteotoxic conditions beyond hyperosmotic stress.

In addition to the proteasomal maintenance of ASK1, the intrarelationship of ASK family proteins should be highlighted for the apoptosis-inducing function of ASK1. Elevated ASK1 protein levels enhanced ASK1-mediated transphosphorylation to ASK3, thereby inhibiting ASK3 inactivation under hyperosmotic stress (Figs. 3, *C* and *D*, and 4*F*). Interestingly, our results suggested that the regulatory effects of ASK1 and ASK3 are reciprocal. In contrast to ASK3, ASK1 is activated in response to hyperosmotic stress (16) (Fig. 4*B*; lanes 5 and 6). ASK1 activation was attenuated as the ratio of ASK1 to ASK3 protein expression decreased (Fig. 3*C*; lanes 5–10), and ASK1 inactivation was even observed in the lowest ASK1-to-ASK3 ratio condition (Fig. 3*C*; lanes 3 and 4). In addition, the overall phosphorylation level of endogenous ASK1 and ASK3 decreased upon hyperosmotic stress (*e.g.*, lanes 1 and 2 in Fig. 1*F*). These results indicate that ASK3 negatively regulates ASK1 activity under hyperosmotic stress. Although we found that the ratio of ASK1 to ASK3 expression levels affects the changes in the activities of both proteins under hyperosmotic stress, further studies are needed to clarify its physiological significance. ASK3 is predominantly expressed in tissues that face extracellular fluid and possess a broad osmolality range, such as the kidneys and stomach, whereas ASK1 is ubiquitously expressed (16, 34). Therefore, cells in the kidney and stomach may acquire increased resistance to hyperosmotic stress-induced apoptosis *via* a low ASK1-to-ASK3 expression ratio. In mouse models of kidney disease, ASK1 is activated and mediates the progression or aggravation of pathological conditions (35, 36). It is of great interest to determine whether the ASK1-to-ASK3 ratio controls any physiological function of ASK1 in kidney disease.

The physiological consequences of the proteasomal regulation of hyperosmotic stress–induced ASK3 inactivation have not been determined. Given that ASK3 inactivation has an essential role in RVI under hyperosmotic stress (17) and that inhibition of RVI is a prerequisite for hyperosmotic stress-induced apoptosis (37), we assumed that insufficient ASK3 inactivation by proteasome inhibition would promote apoptosis under hyperosmotic stress. Although direct evidence has not yet obtained, it is possible that the lack of enhanced apoptosis under hyperosmotic stress in ASK1-knockdown cells treated with proteasome inhibitors (Fig. 4*E*) may result from two overlapping mechanisms: (1) the loss of ASK1’s direct pro-apoptotic function and (2) the restored ability of ASK3 to be inactivated. However, depletion of ASK3 did not attenuate apoptosis under hyperosmotic stress in proteasome-inhibited cells (Fig. S4*A*), suggesting a central role of ASK1 but not ASK3 as the regulator of hyperosmotic stress-induced apoptosis of cells with low proteostasis capacity. ASK3 inhibited hyperosmotic stress-induced SPAK/OSR1 activation when the ASK1 protein level was increased by β-TrCP1/2 depletion (Fig. S4*B*); however, the downstream signaling effects on cellular events should be investigated in future studies.

In summary, we revealed that proteasomal regulation of the ASK1 protein level is important for the modulation of ASK3 signaling and apoptosis under hyperosmotic stress (Fig. 4*F*). Our findings emphasize the role of proteasome activity and the ratio of ASK1 to ASK3 expression in the determination of cell fate under, and possibly beyond, hyperosmotic stress.

## Experimental procedures

### Cell lines and cell culture

Human embryonic kidney 293A (HEK293A) cells (Invitrogen) were cultured in Dulbecco’s modified Eagle’s medium-high glucose (Sigma‒Aldrich, catalog no.: D5796) supplemented with 10% fetal bovine serum and 100 units/ml penicillin G (Meiji Seika, catalog no.: 6111400D2039). Tetracycline-inducible Flag-ASK3-stably expressing HEK293A cells (17) were cultured in Dulbecco’s modified Eagle’s medium supplemented with 10% fetal bovine serum, 2.5 μg/ml blasticidin (Invitrogen, catalog no.: 46-1120 or A1113903) and 50 μg/ml zeocin (Invitrogen, catalog no.: R25001). To induce Flag-ASK3, cells were pretreated with 1 μg/ml tetracycline (Sigma‒Aldrich, catalog no.: T7660) before the assays. All cells were cultured in 5% CO_2_ at 37 °C and verified to be negative for mycoplasma.

### Functional enrichment analysis

Metascape (38) (https://metascape.org) was used to perform Gene Ontology (GO) enrichment analysis and protein-protein interaction analysis of the positive genes identified in the genome-wide siRNA screening for the regulators of ASK3 inactivation under hyperosmotic stress (17). Sixty-three positive genes identified in the second screening were inputted, and the analysis was run with default settings except for background genes, which were set to the genes targeted by the genome-wide siRNA library. GO enrichment analysis was carried out using the following sources: Kyoto Encyclopedia of Genes and Genomes Pathway, GO Biological Processes, Reactome Gene Sets, Canonical Pathways, Cell Type Signatures, CORUM, TRRUST, DisGeNET, PaGenBase, Transcription Factor Targets, WikiPathways, and COVID. The protein-protein interaction analysis was carried out using the following databases: STRING, BioGrid, OmniPath, and InWeb_IM. Only physical interactions identified *via* STRING (physical score >0.132) and BioGrid were used.

### Transfections

Plasmid transfection was performed with polyethylenimine “MAX” (Polysciences, catalog no.: 24765) when HEK293A cells were grown to 60 to 90% confluency, according to a previously described protocol (39) with minor optimization. siRNA transfection for cells at 10 to 40% confluency was carried out by forward (for Figs 4*C*, S1*D*, and S4*B*) or reverse (for other figures) transfection using Lipofectamine️ RNAiMAX (Invitrogen, catalog no.: 13778-150) at a final concentration of 5 to 30 nM for 48 h, according to the manufacturer’s instructions. For experiments in which both the plasmid and siRNA were transfected, siRNA transfection was performed 24 h before plasmid transfection.

### Expression plasmids

The expression plasmids used in this study were constructed by standard molecular biology techniques, and all the constructs were verified by sequencing. For the EGFP-Flag-ASK1 expression plasmid, ASK1 complementary DNA (23) was subcloned and inserted into pcDNA3/GW with an N-terminal EGFP-Flag-tag (18).

### Osmotic stress treatments

Osmotic stress was applied by exchanging the culture medium with osmotic buffer. Iso-osmotic buffer (300 mOsm/kg H_2_O, pH 7.4) contained 130 mM NaCl, 2 mM KCl, 1 mM KH_2_PO_4_, 2 mM CaCl_2_, 2 mM MgCl_2_, 10 mM Hepes, 10 mM glucose, and 20 mM mannitol. The hyperosmotic buffer (400 or 500 mOsm/kg H_2_O, pH 7.4) was the same as the iso-osmotic buffer but contained 120 or 220 mM mannitol, respectively. The 400 or 500 mOsm/kg H_2_O hyperosmotic buffers were created by mixing iso-osmotic buffer and 800 mOsm/kg H_2_O hyperosmotic buffer. Absolute osmolality was verified by Osmomat 030 (Gonotec) to fall within a range from 295 to 315 mOsm for iso-osmotic buffer and ± 25 mOsm/kg H_2_O for the other buffers. In Figures 4, *D* and *E*, S3*A*, and S4*A*, osmotic stress treatment was applied by changing to fresh culture medium supplemented with mannitol and incubating in 5% CO_2_ at 37 °C. For proteasome inhibition, bortezomib (LC Laboratories, catalog no.: B-1408) or MG132 (Enzo Life Sciences, catalog no.: BML-PI102) dissolved in dimethyl sulfoxide (DMSO) was added to the culture medium before osmotic stress treatments. For translation inhibition, cycloheximide (Sigma‒Aldrich, catalog no.: C7698-1G) dissolved in DMSO was added to the culture medium 2 h before osmotic stress treatment.

### Cell lysis and Western blotting

The cells were lysed in lysis buffer (20 mM Tris–HCl [pH 7.5], 150 mM NaCl, 10 mM EDTA, 1% sodium deoxycholate, and 1% Triton X-100) supplemented with 1 mM PMSF, 5 μg/ml leupeptin, and phosphatase inhibitor cocktail (20 mM NaF, 30 mM β-glycerophosphate, 2.5 mM Na_3_VO_4_, 3 mM Na_2_MoO_4_, 12.5 μM cantharidin, and 5 mM imidazole). The cell extracts were clarified by centrifugation, and the supernatant was prepared for further testing by adding an equal volume of 2x SDS sample buffer (80 mM Tris–HCl [pH 8.8], 80 μg/ml bromophenol blue, 28.8% glycerol, 4% SDS, and 10 mM dithiothreitol). After boiling at 98 °C for 3 min, the samples were resolved by SDS-PAGE and electroblotted onto a BioTrace polyvinylidene difluoride membrane (Pall), FluoroTrans W membrane (Pall), or Immobilon-P membrane (Millipore, catalog no.: IPVH00010). The membranes were blocked with 3% or 5% skim milk (Megmilk Snow Brand) in TBS-T (20 mM Tris–HCl [pH 8.0], 137 mM NaCl, and 0.1% Tween-20) and probed with the appropriate primary antibodies diluted with first antibody dilution buffer (TBS-T supplemented with 5% bovine serum albumin [Iwai Chemicals, catalog no.: A001] and 0.1% NaN_3_ [Nacalai Tesque, catalog no.: 312-33]). After replacing and probing the appropriate secondary antibodies diluted with TBS-T containing 3% or 5% skim milk, antibody–antigen complexes were detected on X-ray films (Fujifilm, catalog nos.: 47410-07523, 47410-26615, 47410-07595) or with a FUSION Solo S imaging system (Vazyme) using enhanced chemiluminescence reagents (GE Healthcare). Quantification was performed *via* densitometry using ImageJ (National Institutes of Health) (40). ASK3 phosphorylation level (Fig. 1*E*) was defined as the ratio of phosphorylated protein to total protein. Representative images were adjusted to the appropriate brightness and contrast using the GNU Image Manipulation Program. The specificity of all antibodies has been verified in previous reports (16, 17, 18, 26, 41).

### Immunofluorescence-based assay system for assessing ASK3 phosphorylation level

Tetracycline-inducible Flag-ASK3-stably expressing HEK293A cells were seeded in 96-well plates. The cells were transfected with siRNA or treated with proteasome inhibitors followed by osmotic stress for 10 min. Quantification of ASK3 phosphorylation level was performed using the immunofluorescence-based high content analysis reported by our group (16, 17) with minor modifications. Immunostaining was performed by the following procedure: fixation for 15 min with 4% formaldehyde in PBS, permeabilization for 15 min with 0.2% Triton X-100 in PBS, blocking for 30 min with 5% skim milk in TBS-T, and incubation at 4 °C overnight with the primary antibodies in antibody-dilution buffer. The cells were further incubated at room temperature in the dark for 1 h with the appropriate fluorophore-conjugated secondary antibodies in TBS-T. After staining with Hoechst 33258 dye (Dojindo, catalog no.: 343-07961, 1:1000 dilution) in TBS-T for 5 min, ASK3 phosphorylation level was measured and analyzed by using CellInsight NXT (Thermo Fisher Scientific) with HCS Studio (Thermo Fisher Scientific). The white points, black rectangles, and black lines in each violin plot indicate the median, interquartile range (IQR), and range from the upper quartile plus 1.5xIQR to the lower quartile minus 1.5xIQR, respectively. The area of the violin plot is drawn to be equal across all the experimental conditions. The specificity of all antibodies has been verified in the previous report (17).

### Quantitative RT‒PCR

Cells were seeded in 12-well plates. Two days after seeding, the cells were treated with proteasome inhibitors when the cell density reached 50 to 80%. Total RNA was isolated from cells using Isogen (Wako, catalog no.: 319-90211) and reverse transcribed with ReverTra Ace qPCR RT Master Mix with gDNA Remover (Toyobo, catalog no.: FSQ-301) according to the manufacturer’s instructions. Quantitative PCR was carried out using a LightCycler 96 (Roche) with Kapa SYBR Fast qPCR Master Mix (Kapa Biosystems, catalog no.: KK4602). The expression data were normalized to GAPDH, and the primer sequences used are listed in Table S1.

### Caspase-3 activity assay

Apoptosis induction was measured using the fluorogenic substrate Ac-DEVD-AFC for activated caspase-3 (Cayman, catalog no.: 14459). Cells were lysed with PBS containing 0.1% Triton X-100 after osmotic stress, and the cell lysate was centrifuged at 17,700*g* and 4 °C for 10 min. Lysate samples were individually mixed with reagents in 96-well microplates (25 μl of lysate sample, 49.5 μl of 2× Reaction Buffer [BioVision, catalog no.: 1068], 25 μl of PBS, 5 μl of caspase-3 substrate [1 mM in DMSO], and 0.5 μl of 1 M dithiothreitol [TCI, catalog no.: D1071]). Fluorescence signals were measured at specific wavelengths (excitation/emission = 400/505 nm) using a Varioskan Flash (Thermo Fisher Scientific) after incubation at 37 °C for approximately 90 min. For normalization, the protein concentration in each lysate sample was measured using a DC protein assay (Bio-Rad, catalog nos.: 5000113, 5000114, and 5000115). Caspase-3 activity in each sample was calculated as follows: ([fluorescence intensity of sample – background]/protein concentration of sample [μg/μl]). The relative caspase-3 activity was determined by standardizing the caspase-3 activity of each sample by the caspase-3 activity under the conditions shown in the figure.

### Proteasome activity assay

Cells were seeded in 24-well plates. Two days after seeding, the cells were treated with proteasome inhibitors when the cell density reached 70 to 90%. The cells were lysed in ice-cold buffer containing 25 mM Tris–HCl (pH 7.5), 0.2% Nonidet P-40, 1 mM dithiothreitol, 2 mM ATP, and 5 mM MgCl_2_. The lysate was mixed with the fluorogenic peptide succinyl-Leu-Leu-Val-Tyr-7-amino-4-methylcoumarin (Peptide Institute, catalog no.: 3120-v) in 100 mM Tris–HCl (pH 8.0) at 37 °C. The hydrolysis of succinyl-Leu-Leu-Val-Tyr-7-amino-4-methylcoumarin was measured by a Varioskan Flash (excitation/emission = 360/460 nm).

### Quantification and statistical analysis

The statistical results are expressed as the mean ± SD unless otherwise indicated. No statistical method was utilized to predetermine the sample size. For the quantification of ASK3 phosphorylation level, the Wilcoxon rank sum test was used for comparisons between two groups, and the Kruskal‒Wallis test followed by Dunn’s test was used for comparisons between three groups. For other parameters, an unpaired two-tailed Student’s *t* test was used for comparisons between two groups, and Dunnett’s test or Tukey–Kramer’s test was used for comparisons between three or more groups. The number of samples and sample size are indicated in the figure legends, and no sample was excluded from the statistical tests. Statistical tests were performed using GraphPad Prism, version 7.0c for Mac OS X (GraphPad Software, www.graphpad.com) and R with RStudio (RStudio, Inc, https://www.rstudio.com). *p* < 0.05 was considered to indicate statistical significance. The investigators were not blinded to allocation during the experiments or outcome assessments. The experiments were not randomized.

## Data availability

All the data reported in this study are present in the article, and additional information will be shared by the corresponding author (Kengo Watanabe; ken5watanabe@tokushima-u.ac.jp) upon request.

## Supporting information

This article contains supporting information (1, 2, 3, 4, 5, 6, 7, 8, 9).

## Conflict of interest

The authors declare that they have no conflicts of interest with the contents of this article.
