# Supplementary material for: Proteasomal regulation of ASK family kinases dictates cell fate under hyperosmotic stress
Source: J Biol Chem. 2025 Aug 6;301(9):110566. doi: 10.1016/j.jbc.2025.110566 (PMC12446641; doi:10.1016/j.jbc.2025.110566)
Supplement: Supporting information [file mmc1.pdf]

Supporting Information

Proteasomal regulation of ASK family kinases dictates cell fate under hyperosmotic stress

Xiangyu Zhou[1, †], Kengo Watanabe[1, ‡,*], Kazuhiro Morishita[1], Jun Hamazaki[2], Shigeo Murata[2], Isao Naguro[1, §] & Hidenori Ichijo[1, ¶]

Affiliations

[1]Laboratory of Cell Signaling, Graduate School of Pharmaceutical Sciences, The University of Tokyo, Tokyo 113-0033, Japan

[2]Laboratory of Protein Metabolism, Graduate School of Pharmaceutical Sciences, The University of Tokyo, Tokyo 113-0033, Japan

[†]Present affiliation: Division of Cellular Senescence, Cancer Institute, Japanese Foundation for Cancer Research, Tokyo 135-8550, Japan

[‡]Department of Medical Artificial Intelligence and Data Science, Graduate School of Biomedical Sciences, Tokushima University, Tokushima 770-8503, Japan

[§]Laboratory of Bioresponse Signaling, Faculty of Pharmacy, Juntendo University, Chiba 279-0013, Japan

[¶]Cell Signaling and Stress Responses Laboratory, Advanced Research Institute, Institute of Science Tokyo, Tokyo 101-0062, Japan

*Correspondence: ken5watanabe@tokushima-u.ac.jp (K.W.)

This PDF file includes:

Figure S1 to S4

Table S1 and S2

Figure S1

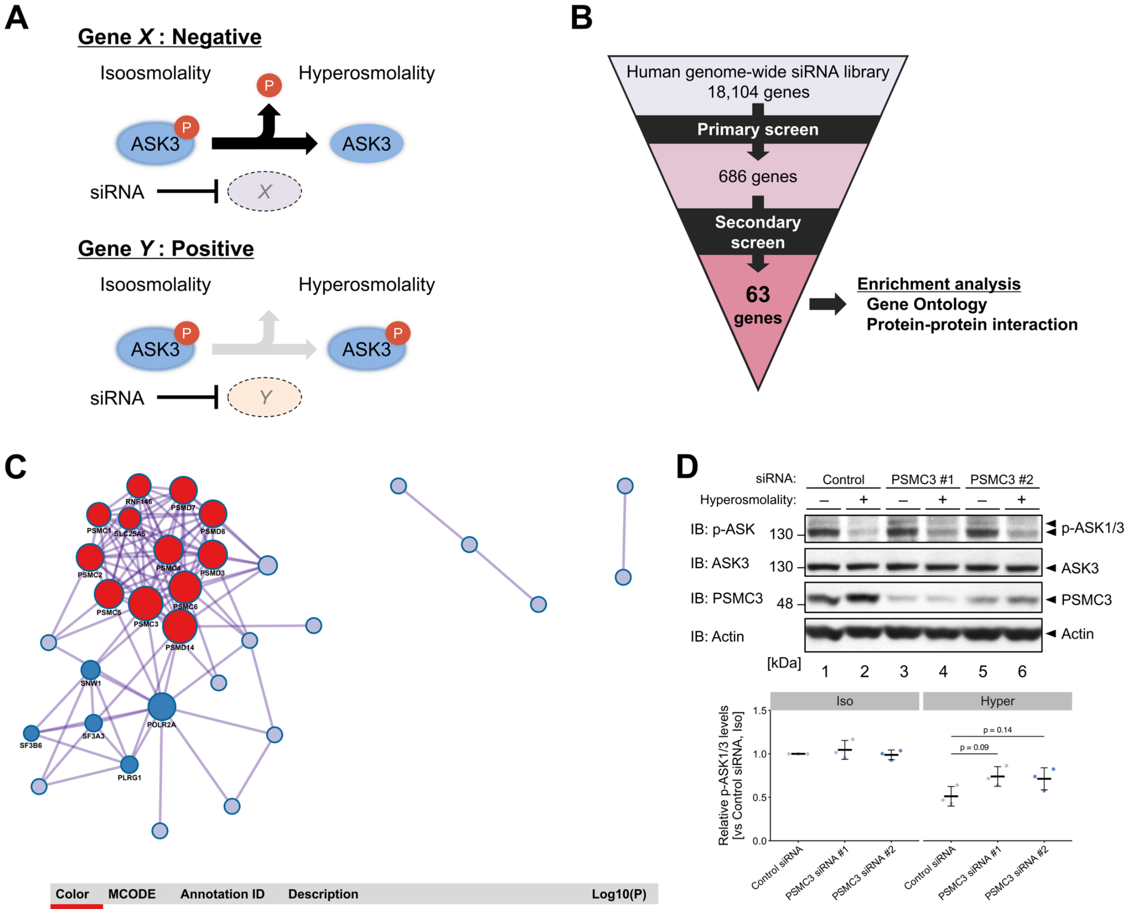

Color	MCODE	Annotation ID	Description	Log10(P)
	MCODE_1	CORUM:32	PA700 complex	–28.9
	MCODE_1	R-HSA-4641257	Degradation of AXIN	–27.1
	MCODE_1	R-HSA-8948751	Regulation of PTEN stability and activity	–26.0
	MCODE_2	R-HSA-72163	mRNA Splicing – Major Pathway	–10.0
	MCODE_2	R-HSA-72172	mRNA Splicing	–9.9
	MCODE_2	R-HSA-72203	Processing of Capped Intron-Containing Pre-mRNA	–9.4

Figure S1. The proteasome as a negative regulator of ASK3 activity under hyperosmotic stress. Related to Figure 1.
(A, B) Rationale (A) and process (B) of the siRNA screening for regulators of ASK3 inactivation under hyperosmotic stress, related to Figure 1A.
(C) Protein–protein interaction (PPI) networks and Molecular Complex Detection (MCODE; [1]) components identified in the positive genes of the secondary screening, related to Figure 1C. Nodes indicate proteins that physically interact with at least one of the other proteins encoded by the positive genes, and edges indicate their PPIs. The densely connected clusters of proteins identified by application of the MCODE algorithm to the PPI network are shown in red or blue. Pathway and process enrichment analyses were applied to each MCODE component, and the three best-scoring terms according to the nominal p value are shown in the lower panel.
(D) Effect of PSMC3 depletion on endogenous p-ASK1/3 levels under hyperosmotic stress, related to Figure 1F. The bottom panel shows the quantification of the western blot data. Individual values and the mean ± SD are presented as points and bars, respectively. $n = 3$ biological replicates. Statistical analysis was performed using Dunnett's test. Iso, 300 mOsm; Hyper, 400 mOsm; 10 min. IB, immunoblotting.

Figure S2

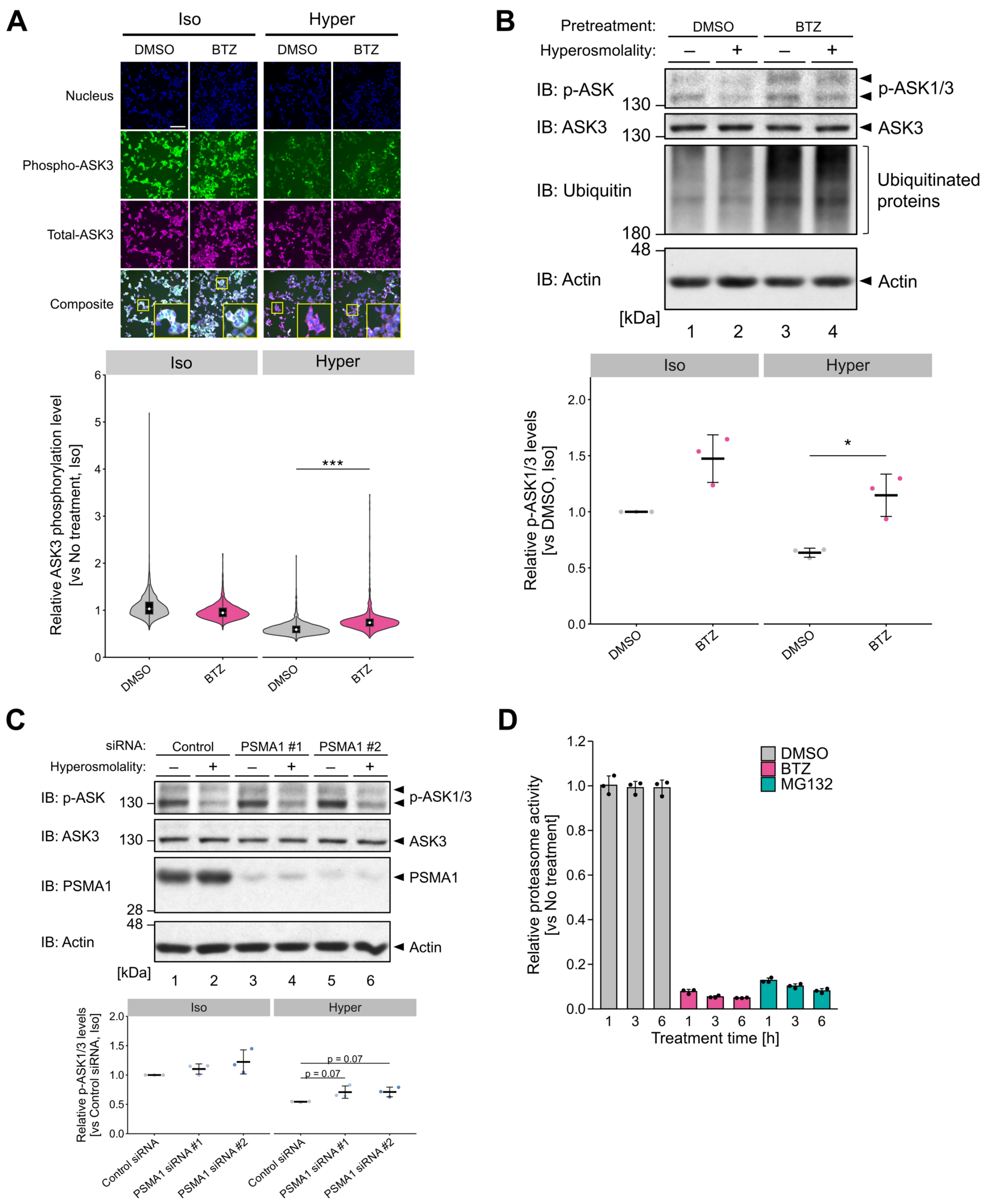

Figure S2. Requirement of proteasome activity for ASK3 inactivation under hyperosmotic stress. Related to Figure 2.

(A) Effect of bortezomib on ASK3 phosphorylation in tetracycline-inducible Flag-ASK3-stably expressing cells, related to Figure 2A. Cells were pretreated with 100 nM bortezomib for 6 h before osmotic stimuli. ASK3

phosphorylation level was measured as described in Figure 1D. The top panel shows immunofluorescence images of nuclei, phospho-ASK3, total-ASK3, and their combination. The bottom panel shows violin plots of ASK3 phosphorylation level in each cell. DMSO: n = 1,724 cells (iso), 1,187 cells (hyper); BTZ: n = 1,316 cells (iso), 1,074 cells (hyper). Representative data from three independent experiments are shown. The white scale bar represents 200 µm.

(B) Effect of bortezomib on endogenous p-ASK1/3 levels, related to Figure 2B. Cells were pretreated with 100 nM bortezomib for 6 h before exposure to osmotic stimuli. The efficacy of proteasome inhibition was confirmed by the accumulation of ubiquitinated proteins. The bottom panel shows the quantification of the western blot data. Individual values and the mean ± SD are presented as points and bars, respectively. n = 3 biological replicates.

(C) Effect of PSMA1 depletion on endogenous p-ASK1/3 levels. The bottom graph shows the quantification of the western blot data. Individual values and the mean ± SD are presented as points and bars, respectively. n = 3 biological replicates.

(D) Effects of proteasome inhibitors on proteasome activity. Cells were treated with 100 nM bortezomib or 10 µM MG132 for the indicated times. Mean ± SD. n = 3 biological replicates.

Statistical analyses were performed using Wilcoxon rank sum test (A), unpaired two-tailed Student's t test (B), and Dunnett's test (C). $^{*}p < 0.05$, $^{***}p < 0.001$. (A, B) Iso, 300 mOsm; Hyper, 400 mOsm; 10 min.

Figure S3

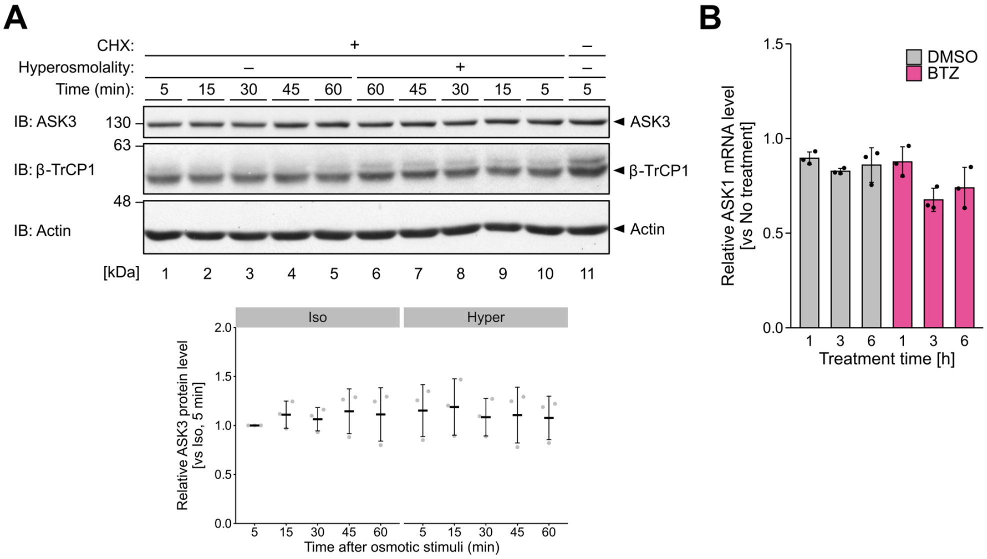

Figure S3. Involvement of ASK1 protein levels in the regulation of ASK3 signaling under hyperosmotic stress. Related to Figure 3.

(A) Effects of hyperosmotic stress on the protein level of ASK3 under conditions of protein synthesis inhibition. Cells were pretreated with 100 μg/mL cycloheximide for 2 h before osmotic stimuli. The bottom graph shows the quantification of the western blot data. Individual values and the mean ± SD are presented as points and bars, respectively. n = 3 biological replicates. The efficiency of protein synthesis inhibition was confirmed by the reduction in β-TrCP1 levels. Iso, culture medium; Hyper, culture medium supplemented with 200 mM mannitol. CHX, cycloheximide; IB, immunoblotting.

(B) Effects of proteasome inhibition on ASK1 mRNA levels, related to Figure 3B. Cells were treated with 100 nM bortezomib for the indicated times. Mean ± SD. n = 3 biological replicates.

Figure S4

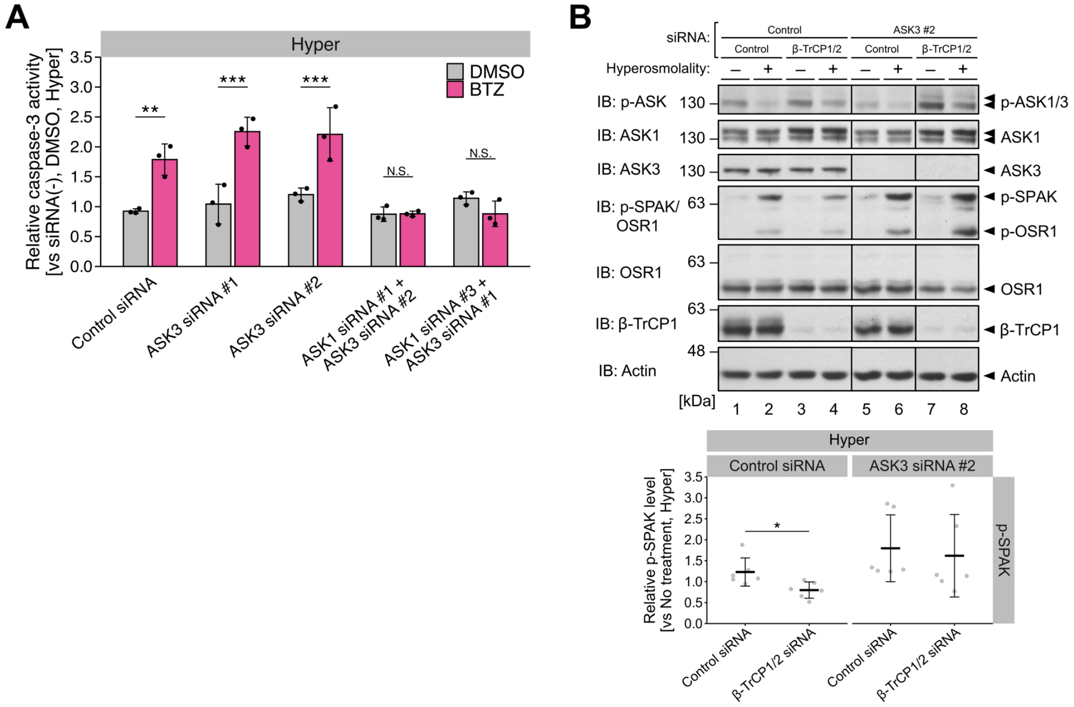

Figure S4. Involvement of ASK3 in hyperosmotic stress-induced apoptosis and SPAK/OSR1 activation during ASK1 accumulation.

(A) Effects of ASK3 and ASK1 depletion on bortezomib-induced overactivation of caspase-3 under hyperosmotic stress, related to Figure 4E. Mean ± SD. n = 3 biological replicates. Hyper, culture medium supplemented with 500 mM mannitol; 12 h.

(B) Requirement of ASK3 for the effects of β-TrCP1/2 depletion on SPAK/OSR1 activation under hyperosmotic stress. The bottom graph shows the quantification of the western blot data. Individual values and the mean ± SD are presented as points and bars, respectively. n = 6 biological replicates. Iso, 300 mOsm; Hyper, 400 mOsm; 10 min. IB, immunoblotting.

Statistical analyses were performed using Tukey–Kramer's test (A) and unpaired two-tailed Student's t test (B). N.S., not significant; $^{*}p < 0.05$, $^{**}p < 0.01$, $^{***}p < 0.001$.

Table S1. Reagents and Resources used in this study.

REAGENT or RESOURCE	SOURCE	IDENTIFIER
Antibodies		
Mouse monoclonal anti-DYKDDDDK tag (Flag; clone 1E6, IB: 1:5,000–1:10,000)	Wako Pure Chemical Industries	Cat#012-22384; RRID: AB_10659717
Mouse monoclonal anti-Actin (Actin; clone AC-40, IB: 1:2,000)	Sigma-Aldrich	Cat#A3853; RRID: AB_262137
Alexa Fluor 488 goat anti-rat IgG (H + L) (IF: 1:200)	Molecular Probes	Cat#A11006; RRID: AB_2534074
Alexa Fluor 594 goat anti-mouse IgG (H + L) (IF: 1:1,000)	Molecular Probes	Cat#A11005; RRID: AB_2534073
Rabbit monoclonal anti-ASK1 (ASK1; clone EP553Y, IB: 1:5,000–1:10,000)	Abcam	Cat#ab45178; RRID: AB_722915
Rat monoclonal anti-ASK3 (ASK3; IB: 1:5,000–1:10,000)	Naguro et al., 2012[2]	N/A
Mouse monoclonal anti-beta-catenin (β-catenin; clone E-5, IB: 1:2,000)	Santa Cruz Biotechnology	Cat#sc-7963; RRID: AB_626807
Rabbit monoclonal anti-β-TrCP (β-TrCP1; clone D13F10, IB: 1:5,000)	Cell Signaling Technology	Cat#4394; RRID: AB_10545763
Mouse monoclonal anti-DYKDDDDK tag (Total-ASK3; clone M2, IF: 1:2,000)	Sigma-Aldrich	Cat#F3165; RRID: AB_259529
Mouse monoclonal anti-OXSR1 (OSR1; clone 2A2-1A2, IB: 1:20,000)	Abnova	Cat#H00009943-M01; RRID: AB_425821
Rabbit polyclonal anti-phospho-ASK (p-ASK1/3; Thr808 in human ASK3 and Thr838 in human ASK1, IB: 1:1,000–1:10,000)	Naguro et al., 2012[2]; Tobiume et al., 2002[3]	N/A
Rabbit polyclonal anti-phospho-SPAK/OSR1 (p-SPAK/OSR1; Thr231 in human SPAK and Thr185 in human OSR1, IB: 1:1,000)	Naguro et al., 2012[2]	N/A
Rat monoclonal anti-phospho-ASK (Phospho-ASK3; Thr808 in human ASK3, clone PA41, IF: 1:500)	Naguro et al., 2012[2]	N/A
Rabbit polyclonal anti-PSMC3 (PSMC3; IB, 1:5,000)	Hamazaki et al., 2007[4]	N/A
Mouse monoclonal anti-Ubiquitin (Ubiquitinated proteins; clone P4D1, IB: 1:10,000)	Santa Cruz Biotechnology	Cat#sc-8017; RRID: AB_628423
Anti-rabbit IgG, HRP-linked (IB: 1:1,000–1:20,000)	Cell Signaling Technology	Cat. #7074; RRID: AB_2099233
Anti-rat IgG, HRP-linked (IB: 1:5,000–1:10,000)	Cell Signaling Technology	Cat. #7077; RRID: AB_10694715
Anti-mouse IgG, HRP-linked (IB: 1:2,000–1:20,000)	Cell Signaling Technology	Cat. #7076; RRID: AB_330924
Chemicals, peptides, and recombinant proteins		
2 × Reaction Buffer	BioVision	Cat#1068
Bortezomib	LC Laboratories	Cat#B-1408
Ac-DEVD-AFC	Cayman	Cat#14459
Dimethyl sulfoxide	Sigma-Aldrich	Cat#D5879
Dithiothreitol	TCI	Cat#D1071
Formaldehyde Solution	Wako Pure Chemical Industries	Cat#064-00406
Hoechst33258	Dojindo	Cat#343-07961
Phosphatase inhibitor cocktail	Watanabe et al., 2018[5]	N/A
MG132	Enzo Life Sciences	Cat#BML-PI102

Opti-MEM	Thermo Fisher Scientific	Cat#31985
Polyethylenimine “MAX”	Polysciences	Cat#24765
Adenosine 5'-Triphosphate Disodium Salt Trihydrate	Wako Pure Chemical Industries	Cat#018-16911
Succinyl-Leu-Leu-Val-Tyr-7-amino-4-methylcoumarin	Peptide Institute	Cat#3120-v
Lipofectamine RNAiMAX	Invitrogen	Cat#133778-150
Tetracycline	Sigma-Aldrich	Cat#T7660
Dulbecco’s Modified Eagle’s Medium - high glucose	Sigma-Aldrich	Cat#D5796
penicillin G	Meiji Seika	Cat#6111400D2039
Cycloheximide	Sigma-Aldrich	Cat#C7698
Experimental models: Cell lines		
Human: HEK293A cells	Invitrogen	N/A
Human: Tetracycline-inducible Flag-ASK3-stably-expressing HEK293A cells	Watanabe et al., 2018[5]	N/A
Oligonucleotides		
Control siRNA (Stealth RNAi Negative Control Medium GC Duplex #2)	Invitrogen	Cat#12935-112
ASK1 siRNA #1 (Stealth RNAi siRNA, target sequence: 5'-GCCAACACUACAGUCAGGAAUUAAU-3')	Invitrogen	Cat#10620312
ASK1 siRNA #2 (Stealth RNAi siRNA, target sequence: 5'-CCUGUGCUAACGACUUGCUUGUUGA-3')	Invitrogen	Cat#10620312
ASK1 siRNA #3 (Stealth RNAi siRNA, target sequence: 5'-UGAAGCUAAGUAGUCUUCUUGGUAA-3')	Invitrogen	Cat#10620312
ASK3 siRNA #1 (Stealth RNAi siRNA, target sequence: 5'-CACCGAAGAGCAGUGCAGUAGAUUU-3')	Invitrogen	Cat#10620312
ASK3 siRNA #2 (Stealth RNAi siRNA, target sequence: 5'-GAGAGGGUUUCUUAAGGCAGGUGAA-3')	Invitrogen	Cat#10620312
b-TrCP1 siRNA (Stealth RNAi siRNA, BTRC-HSS113250, target sequence: 5'-CCAACAUGGGCACAUAAACUCGUAU-3')	Invitrogen	Cat#1299001
b-TrCP2 siRNA (Stealth RNAi siRNA, FBXW11-HSS177307, target sequence: 5'-CCAGCCUGGAAUGUUUGAAAGUGUU-3')	Invitrogen	Cat#1299001
Control siRNA (siGENOME Non-targeting siRNA #4, target sequence: 5'-AUGAACGUGAAUUGCUCAA-3')	Horizon	Cat#D-001210-04
PSMA1 siRNA #1 (siGENOME siRNA, target sequence: 5'-GAUAUGGGCCCUCACAUUU-3')	Horizon	Cat#D-010123-01
PSMA1 siRNA #2 (siGENOME siRNA, target sequence: 5'-GGGCAGGAUUCAUCAAAUU-3')	Horizon	Cat#D-010123-03
PSMC2 siRNA #1 (Stealth RNAi siRNA, PSMC2-HSS108700)	Invitrogen	Cat#1299001
PSMC2 siRNA #2 (Stealth RNAi siRNA, PSMC2-HSS108701)	Invitrogen	Cat#1299001
PSMC2 siRNA #3 (Stealth RNAi siRNA, PSMC2-HSS108702)	Invitrogen	Cat#1299001
PSMC3 siRNA #1 (Stealth RNAi siRNA, PSMC3-HSS108703)	Invitrogen	Cat#1299001
PSMC3 siRNA #2 (Stealth RNAi siRNA, PSMC3-HSS108705)	Invitrogen	Cat#1299001
See Table S2 for primers of qPCR analysis.		
Recombinant DNA		
pcDNA4/TO EGFP-Flag-ASK3 KM	Watanabe et al., 2021[6]	N/A

pcDNA3/GW Flag-ASK1	Takeda et al., 2007[7]	N/A
pcDNA3/GW Flag-ASK1 KM	Takeda et al., 2007[7]	N/A
pcDNA3/GW Flag-ASK3	Naguro et al., 2012[2]	N/A
pcDNA3/GW EGFP-Flag-ASK1	This paper	N/A
Software and algorithms		
GNU Image Manipulation Program (GIMP; ver. 2.8.22)	GIMP Development Team	https://www.gimp.org/
HCS Studio (ver. 6.4.3) or Cellomics vHCS: View (ver. 1.6.2)	Thermo Fisher Scientific	N/A
ImageJ (ver. 1.53k)	Schneider et al., 2012[8]	https://imagej.nih.gov/ij/
Metascape (ver. 3.5)	Zhou et al., 2019[9]	https://metascape.org/
GraphPad Prism 7.0c	GraphPad Software	https://www.graphpad.com/
R (ver. 4.2.2)	R Foundation	https://www.r-project.org/
RStudio (ver. 2022.07.2+576)	RStudio	https://www.rstudio.com/

Table S2. List of the primer sequences used in the qPCR analysis.

Gene	Forward	Reverse
human *ASK1*	5'-CACGTGATGACTTAAAATGCTTG-3'	5'-AGTCAATGATAGCCTTCCACAGT-3'
human *GAPDH*	5'-AGCCACATCGCTCAGACAC-3'	5'-GCCCAATACGACCAAATCC-3'

Reference

1. Bader, G.D., and Hogue, C.W.V. (2003). An automated method for finding molecular complexes in large protein interaction networks. BMC Bioinformatics *4*, 2. 10.1186/1471-2105-4-2.

2. Naguro, I., Umeda, T., Kobayashi, Y., Maruyama, J., Hattori, K., Shimizu, Y., Kataoka, K., Kim-Mitsuyama, S., Uchida, S., Vandewalle, A., et al. (2012). ASK3 responds to osmotic stress and regulates blood pressure by suppressing WNK1-SPAK/OSR1 signaling in the kidney. Nat. Commun. *3*, 1285. 10.1038/ncomms2283.

3. Tobiume, K., Saitoh, M., and Ichijo, H. (2002). Activation of apoptosis signal-regulating kinase 1 by the stress-induced activating phosphorylation of pre-formed oligomer. Journal of Cellular Physiology *191*, 95–104. 10.1002/jcp.10080.

4. Hamazaki, J., Sasaki, K., Kawahara, H., Hisanaga, S.-I., Tanaka, K., and Murata, S. (2007). Rpn10-mediated degradation of ubiquitinated proteins is essential for mouse development. Mol. Cell. Biol. *27*, 6629–6638. 10.1128/MCB.00509-07.

5. Watanabe, K., Umeda, T., Niwa, K., Naguro, I., and Ichijo, H. (2018). A PP6-ASK3 Module Coordinates the Bidirectional Cell Volume Regulation under Osmotic Stress. Cell Rep. *22*, 2809–2817. 10.1016/j.celrep.2018.02.045.

6. Watanabe, K., Morishita, K., Zhou, X., Shiizaki, S., Uchiyama, Y., Koike, M., Naguro, I., and Ichijo, H. (2021). Cells recognize osmotic stress through liquid-liquid phase separation lubricated with poly(ADP-ribose). Nat. Commun. *12*, 1353. 10.1038/s41467-021-21614-5.

7. Takeda, K., Shimozono, R., Noguchi, T., Umeda, T., Morimoto, Y., Naguro, I., Tobiume, K., Saitoh, M., Matsuzawa, A., and Ichijo, H. (2007). Apoptosis signal-regulating kinase (ASK) 2 functions as a mitogen-activated protein kinase kinase kinase in a heteromeric complex with ASK1. J. Biol. Chem. *282*, 7522–7531. 10.1074/jbc.M607177200.

8. Schneider, C.A., Rasband, W.S., and Eliceiri, K.W. (2012). NIH Image to ImageJ: 25 years of image analysis. Nat. Methods *9*, 671–675. 10.1038/nmeth.2089.

9. Zhou, Y., Zhou, B., Pache, L., Chang, M., Khodabakhshi, A.H., Tanaseichuk, O., Benner, C., and Chanda, S.K. (2019). Metascape provides a biologist-oriented resource for the analysis of systems-level datasets. Nat. Commun. *10*, 1523. 10.1038/s41467-019-09234-6.
